# Genetic variation in the TNF receptor-associated factor 6 gene is associated with susceptibility to sepsis-induced acute lung injury

**DOI:** 10.1186/1479-5876-10-166

**Published:** 2012-08-17

**Authors:** Zhenju Song, Chenling Yao, Jun Yin, Chaoyang Tong, Duming Zhu, Zhan Sun, Jinjun Jiang, Mian Shao, Yaping Zhang, Zhi Deng, Zhengang Tao, Si Sun, Chunxue Bai

**Affiliations:** 1 Department of Emergency Medicine, Zhongshan Hospital, Fudan University, Shanghai, People’s Republic of China; 2 Department of Anesthesiology, Zhongshan Hospital, Fudan University, Shanghai, People’s Republic of China; 3 Department of Pulmonary Medicine, Zhongshan Hospital, Fudan University, Shanghai, People’s Republic of China

**Keywords:** Acute lung injury, Genetic variation, TRAF6, TLR signaling pathway

## Abstract

**Background:**

Recent studies showed that overwhelming inflammatory response mediated by the toll-like receptor (TLR)-related pathway was important in the development of acute lung injury (ALI). The aim of this study was to determine whether common genetic variation in four genes of the TLR signaling pathway were associated with sepsis-induced ALI susceptibility and risk of death in Chinese Han population.

**Methods:**

Fourteen tag single nucleotide polymorphisms (tagSNPs) in *MyD88*, *IRAK1*, *IRAK4* and *TRAF6* were genotyped in samples of sepsis-induced ALI (n = 272) and sepsis alone patients (n = 276), and tested for association in this case-control collection. Then, we investigated correlation between the associated SNP and the mRNA expression level of the corresponding gene. And we also investigated correlation between the associated SNP and tumor necrosis factor alpha (TNF-α) as well as interleukin-6 (IL-6) concentrations in peripheral blood mononuclear cells (PBMCs) exposed to lipopolysaccharides (LPS) *ex vivo*. The mRNA expression level was determined using real-time quantitative Polymerase Chain Reaction (PCR) assays, and concentrations of TNF-α and IL-6 were measured by enzyme-linked immunosorbent assay (ELISA).

**Results:**

The association analysis revealed that rs4755453, an intronic SNP of *TRAF6*, was significantly associated with susceptibility to sepsis-induced ALI. The C allele frequency of rs4755453 in the sepsis alone group was significantly higher than that in the sepsis-induced ALI group (*P* = 0.00026, odds ratio (OR) = 0.52, 95% confidence interval (CI) 0.37–0.74). These associations remained significant after adjustment for covariates in multiple logistic regression analysis and for multiple comparisons. *TRAF6* mRNA expression levels in PBMCs from homozygotes of the rs4755453G allele were significantly higher than that in heterozygotes and homozygotes of the rs4755453C allele at baseline (*P* = 0.012 and *P* = 0.003, respectively) as well as after LPS stimulation (*P* = 0.009 and *P* = 0.005). Moreover, the concentrations of TNF-α and IL-6 in cell culture supernatants were also significantly higher in the subjects with rs4755453GG genotype than in subjects with CG and CC genotype. None of the 14 tagSNPs showed associations with risk of death and severity among ALI cases.

**Conclusions:**

Our findings indicated that common genetic variants in *TRAF6* were significantly associated with susceptibility to sepsis-induced ALI in Chinese Han population. This was the first genetic evidence supporting a role for *TRAF6* in ALI.

## Background

Acute lung injury (ALI) and its more severe form, the acute respiratory distress syndrome (ARDS), are characterized by increased inflammatory cytokine expression and cell infiltration into the lungs, non-cardiogenic pulmonary edema, and diffuse alveolar damage that culminates in respiratory failure [1]. ALI remains an important cause of death in the intensive care unit (ICU) and few specific therapies are available [2]. The causes of ALI are numerous (e.g., pneumonia, sepsis, aspiration, trauma and pancreatitis), but the reasons why certain individuals develop lung injury in response to these stimuli and others do not are not well understood. There was abundant evidence in the literature that gene-host and gene-environment interactions might play a large role in the morbidity and mortality associated with this syndrome [3,4]. A number of recent studies revealed that genetic variation might confer risk of developing ALI or influence ALI mortality [5-19]. To date, genetic variants in more than 20 genes were found to be associated with ALI and ALI-related outcomes.

Although the exact mechanism of ALI/ARDS remained incompletely understood, it was well established that overwhelming inflammation was a fundamental component of the pathophysiology [2]. TLRs, a family of immune receptors, are described to be involved in the recognition of both pathogen-associated molecular patterns (PAMPs) and damage-associated molecular patterns (DAMPs). TLR signaling pathway is regulated by TIR domain-containing adaptors. Upon ligand binding to TLR, the adaptor molecule Myeloid differentiation factor 88 (MyD88) is recruited to TLR complex as a dimer. Then MyD88 recruits interleukin-1 receptor-associated kinase 1 (IRAK1), IRAK4, and TNF receptor-associated factor 6 (TRAF6), which results in the activation of NF-κB and production of inflammatory cytokines [20]. MyD88, as an adaptor molecule of TLRs, plays an important role in LPS-induced inflammation. MyD88 knockout mice showed no responses to LPS in terms of macrophage production of inflammatory mediators [21]. Both IRAK1 and IRAK4 contain an N-terminal death domain, which is responsible for interaction with MyD88, and have an essential role in the activation of NF-κB and MAPK. Animal studies found that IRAK-4 or IRAK1 knockout mice have an impaired response to various microbial components [22,23]. TRAF6 exhibits various functions in regulating adaptive and innate immunity, and cell apoptosis [24]. The immune response initiated by TLR signaling pathway is an important mechanism in defense against pathogenic microorganisms and maintenance of tissue integrity. However, prolonged and excessive activation of TLR signaling pathway causes the overproduction of inflammatory cytokines and contributes to tissue or organ injury [25]. There was increasing evidence that excessive inflammation mediated by the TLR-related pathway might contribute to the morbidity and mortality of ALI [26].

Several SNPs within genes encoding the activating TLR signaling pathway were reported to influence the production of inflammatory cytokines and be associated with susceptibility to ALI and sepsis. Two variants in the TLR4 gene, *D299G* (rs4986790) and *T399I* (rs4986791), were associated with susceptibility to sepsis [27]. Recently, *TLR1* -7202A/G (rs5743551) was reported to be correlated with hyper-inflammatory responses to PAMPs and associated with increased susceptibility to sepsis-induced ALI and organ dysfunction [28,29]. Two studies showed that a haplotype in *IRAK-1,* which increased nuclear levels of NF-κB, was related to severity and mortality of sepsis [30,31]. Rs8177374 (S180L), located in TIRAP on chromosome 11q24.2, was associated with the risk of invasive pneumococcal disease and septic shock [32,33]. Pino-Yanes et al. found that four common variants (rs1732888, rs1732887, rs1732886 and rs10506481) of the IRAK3 gene were associated with ALI development during severe sepsis [13]. Our latest studies also found two SNPs (rs8177375 and rs595209) within *TIRAP* were associated with the susceptibility to sepsis-induced ALI [34]. However, the role of genetic variation within downstream components of the TLR signaling pathway on ALI development and mortality remained largely unexplored.

Given the importance of exaggerated inflammatory response in the pathogenesis of ALI and the pivotal role of TLR signaling pathway in inflammatory response, we hypothesized that genetic variation in the TLR signaling pathway genes might be associated with susceptibility and outcome of ALI. To test this hypothesis, we conducted a case-control study using tag SNP approach to investigate the association of variants in *MyD88*, *IRAK1*, *IRAK4* and *TRAF6* with susceptibility and outcome of sepsis-induced ALI in Chinese Han population. In addition, we performed functional evaluation of the associated SNP.

## Materials and methods

### Study design and enrollment

Definitions of sepsis and ALI/ARDS were in accordance with the American College of Chest Physicians/Society of Critical Care Medicine Consensus Conference [35] and the American-European Consensus Conference statements [36]. All sepsis subjects enrolled had either severe sepsis or septic shock. All patients were selected from the Emergency, Surgical and Respiratory ICU at Zhongshan Hospital, Fudan University. Exclusion criteria included age < 18 years, pregnancy, diffuse alveolar hemorrhage, severe chronic respiratory disease, directive to withhold intubation, severe chronic liver disease (defined as a Child–Pugh score of > 10), malignancy, using of chronic high-dose immunosuppressive therapy (steroids with equivalent prednisone ≥ 0.5 mg/kg per day or cytotoxic agents for immunologic disorders) and AIDS patients. All sepsis patients were screened daily for ALI/ARDS development and those who fulfilled the AECC criteria for ALI/ARDS were considered as ALI cases, which included ALI and ARDS patients; whereas those patients who did not develop ALI/ARDS during hospital stay were considered as sepsis alone patients. Clinical and demographic data at baseline, including Acute Physiology and Chronic Health Evaluation (APACHE) II scores, organ failure, previous health status, hospital and ICU mortality were obtained after the patient met inclusion criteria. Part of the patients included in the present study overlapped with that in our previous study [34].

This study was approved by the Ethic Committee of Zhongshan Hospital, Fudan University, Shanghai, China (Record no: 2006-23). Informed consent was obtained from subjects or from their legal surrogates before enrollment. Recent analyses by Genome-wide SNP variation have shown that the central Han Chinese could be regarded as one single homogenous population [37,38]. To reduce the potential confounding from ethnic backgrounds, we only enrolled people with self-reported origin of central Han Chinese, including indigenous people from Zhejiang Province, Jiangsu Province, Anhui Province and Shanghai.

### SNPs selection and genotyping

A total of four candidate genes involved in TLR signaling pathway were selected according to the known biological activity: *MyD88IRAK1IRAK4* and *TRAF6*. TagSNPs were selected on the basis of the Chinese Han in Beijing data from the HapMap project phase II (http://www.hapmap.org/) [39]. The tagSNPs covered the gene regions and up to 1 kb of 3’ as well as 5’ flanking regions of the candidate genes. In total, 14 tagSNPs in the four genes were selected by tagger implemented in Haploview using the following tagging criteria: pairwise tagging of the HapMap population with r^2^ of at least 0.8 and a minor allele frequency of at least 5%. Location and characterization of all tested tagSNPs were listed in Table 1. Among the 14 tagSNPs, two were non-synonymous.

**Table 1:** Characteristics of the genotyped SNPs in the genes of TLR signaling pathway

**Gene**	**SNP**	**Location**	**Major/minor allele**	**HWE*P*value**
MyD88	rs6853	3′ UTR	A/G	0.35
	rs7744	3′ UTR	A/G	0.48
IRAK1	rs1059703	exon	C/T	0.45
IRAK4	rs3794262	intron	A/T	0.48
	rs4251429	intron	G/C	0.97
	rs4251545	exon	G/A	0.75
	rs4251569	intron	C/T	0.47
	rs4251513	intron	C/G	0.94
	rs4251466	intron	C/T	0.39
	rs4251431	intron	G/T	0.37
	rs1461567	intron	C/T	0.11
TRAF6	rs540386	intron	C/T	1.00
	rs4755453	intron	G/C	0.32
	rs5030493	intron	T/A	1.00

SNP, single nucleotide polymorphism; HWE, Hardy-Weinberg equilibrium; UTR, untranslated region.

Genomic DNA was extracted from whole blood with a FlexiGene DNA Kit (Qiagen, Hilden, Germany) in accordance with the protocol of the manufacturer. Twelve tagSNPs in *IRAK1*, *IRAK4* and *TRAF6* were genotyped on the GenomeLab SNPstream high-throughput 12-plex genotyping platform (Beckman Coulter, Fullerton, CA) following the manufacturer’s instructions. The primers for PCR and single base extension were designed by Beckman Coulter Autoprimer software and were shown in Table S1 in Additional file 1. Two tagSNPs in *MyD88* were genotyped by direct sequencing. The sequencing reactions were performed using Applied Biosystems BigDye (version 3.1) chemistry (Applied Biosystem, Foster City, CA, USA), and the sequences were resolved using an ABI 3730 Genetic Analyzer. The primers and PCR protocols used were shown in Table S2 in Additional file 1. Genotyping was carried out blind to case–control status. One duplicate sample was added to each 96-well sample plate for quality assurance and quality control validation of inter-plate discordance, and we placed an extra 10 duplicates into our sample set in order to test for experiment-wide discordance. The data completion rate was 99.2%.

### Isolation and stimulation of cells from healthy subjects

PBMCs were derived from 90 unrelated healthy Chinese Han volunteers using Ficoll gradient density centrifugation method. Isolated PBMCs were plated at a density of 1 x 10^6^ cells/ml in 24-well plates and cultured in RPMI 1640 medium with 10% FBS at 37°C with 5% CO_2_. The cells were then incubated for 6 hours in presence or absence of 100 ng/ml Escherichia coli 0111:B4 LPS (Sigma, USA). After incubation, supernatants and cell pellets were harvested and stored at −80°C until use.

### RNA purification and TRAF6 mRNA expression analysis

Total RNA was extracted using RNeasy Mini kit (Qiagen, Hilden, Germany). 100 ng RNA was used for cDNA synthesis using a High Capacity cDNA Reverse Transcription Kit (Applied Biosystems) according to the manufacturer’s protocol. Quantitative RT-PCR was performed using SYBR Green (TaKaRa) on an ABI PRISM 7900 Sequence Detector (Applied Biosystems, USA) with SDS 2.1 software. Each reaction was performed in triplicate, with final calculations resulting from means of triplicate wells. The ΔΔCq method was used to determine the difference for the mean expression levels of *TRAF6* between study subjects with different genotypes of rs4755453. For each individual, the relative expression level ΔCq (Cq T - Cq E) of *TRAF6* was normalized with *GAPDH* and then transformed into relative quantity using the RQ formula (RQ = 2^-ΔΔCq^ , where ΔΔCq is for the individual and ΔCq is the calibrator). The primers for *TRAF6* were: forward 5′- AGGGACCCAGCTTTCTTTGT-3′ and reverse 5′- GCCAAGTGATTCCTCTGCAT-3′. The primers for *GAPDH* were: forward 5′-TGAAGGTCGGAGTCAACGGATTTGGT-3′ and reverse 5′-CATGTGGGCCAT GAGGTCCACCAC-3′.

### Measurement of TNF-α and IL-6 levels

Concentrations of TNF-α and IL-6 in culture supernatants were determined by human ELISA kit (R&D Systems, USA) according to the manufacturer’s protocol.

### Statistical analysis

The genotype data was analyzed for deviations from Hardy-Weinberg equilibrium by the Haploview v4.1 software [40]. Univariate analysis was performed using χ^2^ test for categorical variables and Student’s *t*-test for continuous variables as appropriate. Variables with *P* < 0.2 were entered into a logistic-regression model using a backward-selection algorithm. The final model included gene effect, variables from backward elimination, and clinically relevant variables such as age, gender, organ failure, APACHE II score, transfusion of PRBC, number of PRBC transfused and infection site. The differences of allele and genotype distributions between case and control groups were compared using χ^2^-test or Fisher’s exact test when appropriate. P values for genotypic distributions were calculated using the global genotype test. Allele frequencies of cases and controls were used to calculate the OR and the 95% CI. The Bonferroni method was used to correct for multiple comparisons where applicable. A two tailed *P*-value of < 0.05 was considered statistically significant, whereas a value of corrected *P* < (0.05/number of tests), was considered significant after Bonferroni correction. *IRAK1* is X chromosome linked. We used logistic regression method including sex as a covariate to analysis the association with *IRAK1* SNPs. For the *IRAK1* SNPs, males were coded as homozygote females in the logistic regression analysis. Differences in relative mRNA expression, TNF-α and IL-6 levels among three genotypes were evaluated by Kruskal-Wallis test. When a significant difference was obtained in Kruskal-Wallis test, Mann-Whitney U test was used to identify specific group differences. The software used for statistical calculations was SPSS 15.0 (SPSS Inc., Chicago, IL, USA) unless specified.

## Results

### Characteristics of the study population

From February 2006 to December 2010, a total of 272 sepsis-induced ALI (66 ALI and 206 ARDS patients) and 276 sepsis alone patients were enrolled in this study. The baseline characteristics and clinical data of all subjects were shown in Table 2. The mean age was 64.1 years for sepsis-induced ALI patients and 63.3 years for sepsis alone patients (*P* > 0.05). The proportion of male was 61.4% in ALI patients and 59.1% in sepsis alone patients (*P* > 0.05). ALI patients had higher average APACHE II scores and mortality ratio, and more organ failures than sepsis alone patients (*P* < 0.01).

**Table 2:** Demographic and clinical characteristics of the study subjects

**Characteristic**	**Sepsis alone patients**	**Sepsis-induced ALI patients**	***P*Value**
Total no.	276	272	N.A
Patient age	63.3 ± 13.8	64.1 ± 12.5	0.65
Male patients	163 (59.1%)	167 (61.4%)	0.58
BMI	21.8 ± 5.4	22.1 ± 8.1	0.14
Smoker	115 (41.7%)	113 (41.5%)	0.98
Liver cirrhosis	7 (2.5%)	8 (2.9%)	0.80
Diabetes	37 (13.4%)	32 (11.8%)	0.56
30-d mortality	110 (39.9%)	142 (52.2%)	0.004
APACHE II score	14.7 ± 7.2	19.6 ± 3.4	<0.001
Organ failure	1.9 ± 0.2	3.1 ± 0.2	<0.001
Transfusion of PRBC	75 (27.2%)	105 (38.6%)	0.004
Number of PRBC transfused	1.2	1.6	0.012
Infection site			
Pulmonary source	158 (57.2%)	179 (65.8%)	0.039
Extrapulmonary source	118 (42.8%)	93 (34.2%)	0.039

ALI, acute lung injury; N.A, not applicable; BMI, body mass index; APACHE II, acute physiology and chronic health evaluation; PRBC, packed red blood cells.

### Association analyses of *MyD88*, *IRAK1*, *IRAK4* and *TRAF6* polymorphisms with susceptibility to sepsis-induced ALI

The allele and genotype distributions of all tagSNPs in sepsis-induced ALI and sepsis alone patients were listed in Table 3. The genotyping success rates of all tested SNPs ranged from 98% to 99%. All the genotyped SNPs did not diverse significantly from Hardy-Weinberg equilibrium. The HWE p-values for all tested SNPs in controls and cases were shown in Table 1. Single locus analysis showed that rs4755453, an intronic SNP of *TRAF6*, was significantly associated with risk of ALI, whereas other 13 tagSNPs showed no associations. The C allele frequency of rs4755453 in the sepsis alone group was significantly higher than that in the ALI group (*P* = 0.00026, OR = 0.52, 95% CI 0.37–0.74), which remained significantly after Bonferroni correction (*P* = 0.0036, corrected for 14 SNPs tested). Furthermore, in multivariate analyses after adjustment for age, gender, organ failure, APACHE II score, transfusion of PRBC, number of PRBC transfused and infection site, rs4755453 was still significantly associated with susceptibility to sepsis-induced ALI (*P*_adj_ = 0.0012, OR_adj_ = 0.71, 95% CI 0.58–0.82). The genotype distributions of rs4755453 were significantly different between ALI group and sepsis alone group (*P* = 0.00046), and the significance remained present in a multivariate analysis controlling for covariates (*P*_adj_ = 0.0021) and after Bonferroni correction (*P* = 0.0064, corrected for 14 SNPs tested) (Table 3). Assuming the prevalence of 0.01 and using a significance level of 0.05, our study had over 99% power to detect association with rs4755453 (MAF of 14%) in 272 sepsis-induced ALI vs. 276 sepsis alone patients. These results implicated the minor allele C has a protective effect against the development of sepsis-induced ALI in Chinese Han population.

**Table 3:** **Association analysis of genetic variation in *TRAF6, MyD88,IRAK4 *and *IRAK1 *between sepsis-induced ALI and sepsis alone patient**

**Gene**	**SNP**	**Sepsis alone**	**Sepsis-induced ALI**	**Allelic Comparison**				**Genotypic Comparison**	
				***P***	***P***_**adj**_	**OR (95% CI)**	**OR**_**adj**_**(95% CI)**	***P***	***P***_**adj**_
TRAF6	rs540386			0.24	0.42	0.69 (0.37–1.28)	0.72 (0.42–1.47)	0.23	0.42
	CC	248 (90.8%)	250 (93.6%)						
	TC	25 (9.2%)	17 (6.4%)						
	C	521 (95.4%)	517 ( 96.8%)						
	T	25 (4.6%)	17 (3.2%)						
	rs4755453			0.00026	0.0012	0.52 (0.37–0.74)	0.71 (0.58–0.82)	0.00046	0.0021
	GG	181 (66.5%)	215 (80.2%)						
	CG	81 (29.8%)	51 (19%)						
	CC	10 (3.7%)	2 (0.75%)						
	G	446 (81.4%)	481 (89.7%)						
	C	98 (18.6%)	55 (10.3%)						
	rs5030493			0.23	0.36	0.78 (0.53–1.17)	0.69 (0.42–1.21)	0.15	0.24
	AA	214 (79%)	218 (82%)						
	TA	53 (19.6%)	48 (18%)						
	TT	4 (1.5%)	0						
	A	481 (88.8%)	484 (91%)						
	T	61 (11.2%)	48 (9%)						
MyD88	rs6843			0.98	0.81	1.01 (0.50–2.04)	1.04 (0.62–2.12)	0.98	0.82
	AA	256 (94.1%)	254 (94.1%)						
	AG	16 (5.9%)	16 (5.9%)						
	A	528 (97.1%)	524 (97%)						
	G	16 (2.9%)	16 (3%)						
	rs7744			0.73	0.75	1.04 (0.82–1.33)	1.03 (0.81–1.35)	0.91	0.92
	AA	90 (33.3%)	87 (32.5%)						
	AG	146 (54.1%)	144 (53.7%)						
	GG	34 (12.6%)	37 (13.8%)						
	A	326 (60.4%)	318 (59.3%)						
	G	214 (39.6%)	218 (40.7%)						
IRAK4	rs3794262			0.38	0.65	1.16 (0.84–1.61)	1.02 (0.86–1.83)	0.33	0.46
	AA	198 (72.8%)	182 (68.2%)						
	TA	68 (25%)	81 (30.3%)						
	TT	6 (2.2%)	4 (1.5%)						
	A	464 (85.3%)	445 (83.3%)						
	T	80 (14.7%)	89 (16.7%)						
	rs4251429			0.37	0.58	1.26 (0.76–2.06)	1.13 (0.82–1.93)	0.26	0.52
	GG	242 (89.3%)	233 (86.3%)						
	GC	28 (10.3%)	37 (13.7%)						
	CC	1 (0.4%)	0						
	G	512 (94.5%)	503 (93.1%)						
	C	30 (5.5%)	37 (6.9%)						
	rs4251545			0.41	0.46	1.18 (0.79–1.77)	1.13 (0.65–1.67)	0.51	0.65
	GG	228 (83.5%)	215 (80.2%)						
	GA	41 (15%)	50 (18.7%)						
	AA	4 (1.5%)	3 (1.1%)						
	G	497 (91%)	480 (89.6%)						
	A	49 (9%)	56 (10.4%)						
	rs4251569			0.65	0.52	1.09 (0.76–1.56)	1.24 (0.79–2.01)	0.55	0.32
	CC	207 (77.2%)	200 (74.6%)						
	CT	56 (20.9%)	65 (24.3%)						
	TT	5 (1.9%)	3 (1.1%)						
	C	470 (87.7%)	465 (86.8%)						
	T	66 (12.3%)	71 (13.2%)						
	rs4251513			0.41	0.47	0.90 (0.70–1.15)	0.97 (0.82–1.26)	0.68	0.81
	CC	103 (37.7%)	111 (41.4%)						
	CG	128 (46.9%)	119 (44.4%)						
	GG	42 (15.4%)	38 (14.2%)						
	C	334 (61.2%)	341 (63.6%)						
	G	212 (38.8%)	195 (36.4%)						
	rs4251466			0.59	0.52	1.12 (0.74–1.69)	1.16 (0.82–1.75)	0.09	0.07
	CC	226 (84%)	216 (80.6%)						
	CT	38 (14.1%)	51 (19%)						
	TT	5 (1.9%)	1 (0.4%)						
	C	490 (91.1%)	483 (90.1%)						
	T	48 (8.9%)	53 (9.9%)						
	rs4251431			0.79	0.82	0.95 (0.63–1.43)	0.84 (0.76–1.32)	0.39	0.52
	GG	224 (82.7%)	221 (82.5%)						
	GT	43 (15.9%)	46 (17.2%)						
	TT	4 (1.5%)	1 (0.4%)						
	G	491 (90.6%)	488 (91%)						
	T	51 (9.4%)	48 (9%)						
	rs1461567			0.78	0.81	1.04 (0.81–1.32)	1.02 (0.92–1.25)	0.11	0.23
	CC	80 (29.4%)	88 (33%)						
	TC	136 (50%)	110 (41.2%)						
	TT	56 (20.6%)	69 (25.8%)						
	C	296 (54.4%)	286 (53.6%)						
	T	248 (45.6%)	248 (46.4%)						
IRAK1	rs1059703			0.26	0.73	0.70 (0.28–1.74)^1^	0.78 (0.38–1.69)	–	–
	CC/C-	230 (84.6%)	220 (82.7%)						
	CT	30 (11%)	38 (14.3%)						
	TT/T-	12 (4.4%)	8 (3%)						

SNP, single nucleotide polymorphism; ALI, acute lung injury; OR, odds ratio; CI, confidence interval.

Data were no. (%) of subjects. *P* was determined using the chi-square test. *P*_*adj*_ and OR_adj_ came from multivariate logistic regression.

A *P*-value of < 0.0036 (0.05/14) was considered statistically significant after Bonferroni correction.

^1^ The p value was calculated using logistic regression method including sex as a covariate, in which males were coded as homozygote females.

### Association analyses of *MyD88*, *IRAK1*, *IRAK4* and *TRAF6* polymorphisms with ALI severity and mortality

We next tested for associations between all test SNPs and 30-day mortality. The overall 30-day mortality among genotyped subjects with ALI was 52.2%. We did not find association between *MyD88*, *IRAK1*, *IRAK4* and *TRAF6* variants and 30-day mortality in the ALI cohort in either the unadjusted or adjusted models (Additional file 1: Table S3). Then, we made an association analysis in ALI patients to explore the relation of *MyD88*, *IRAK1*, *IRAK4* and *TRAF6* variants and ALI severity. The allele and genotype frequencies of all tagSNPs were not significantly different between ALI and ARDS groups (Additional file 1: Table S4). Moreover, no significant difference was found between *MyD88*, *IRAK1*, *IRAK4* and *TRAF6* variants and P/F ratio (*P* > 0.05). Taken together, our results suggested that the variation in *MyD88*, *IRAK1*, *IRAK4* and *TRAF6* had no effect on the severity and mortality of ALI.

### Association analyses of *TRAF6* mRNA expression levels with rs4755453 genotype

**Figure 1:**
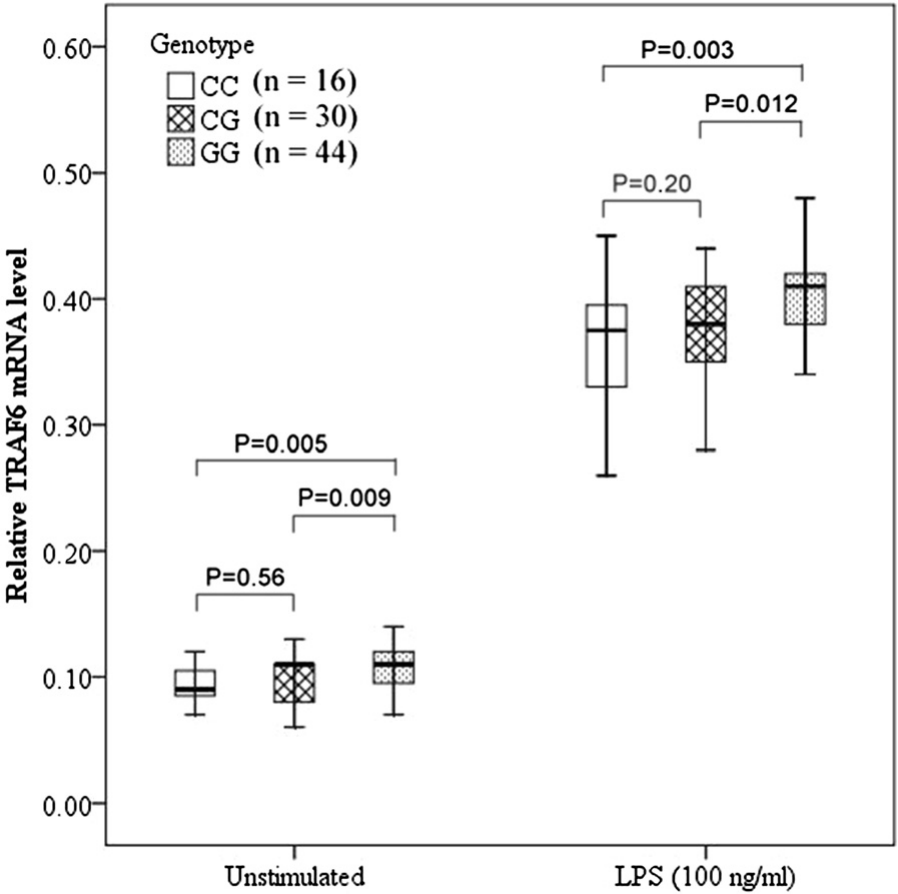
**Association of *TRAF6 *mRNA expression levels and rs4755453 genotype.** Expression levels of *TRAF6* mRNA in PBMCs were normalized with *GAPDH* expression and expressed as the median, interquartile range and extremes. The mRNA expression levels were significantly different among GG, GC and CC genotypes both at baseline (*P* = 0.002) and under the LPS-stimulated condition (*P* = 0.004).

To determine the association between rs4755453 genotype and *TRAF6* mRNA levels in PBMCs, we selected 44 subjects with rs4755453GG genotype; 30 subjects with GC genotype and 16 subjects with CC genotype, who were matched for age and sex. As shown in Figure 1, the *TRAF6* mRNA expression in PBMCs was significantly higher in GG homozygotes compared with both GC heterozygotes and CC homozygotes both at baseline (*P* = 0.012 and *P* = 0.003, respectively) and after stimulation with LPS for 6 hrs (*P* = 0.009 and *P* = 0.005, respectively), whereas the difference between the GC and CC groups was not statistically significant.

### Association analyses of TNF-α and IL-6 levels with rs4755453 genotype

**Figure 2:**
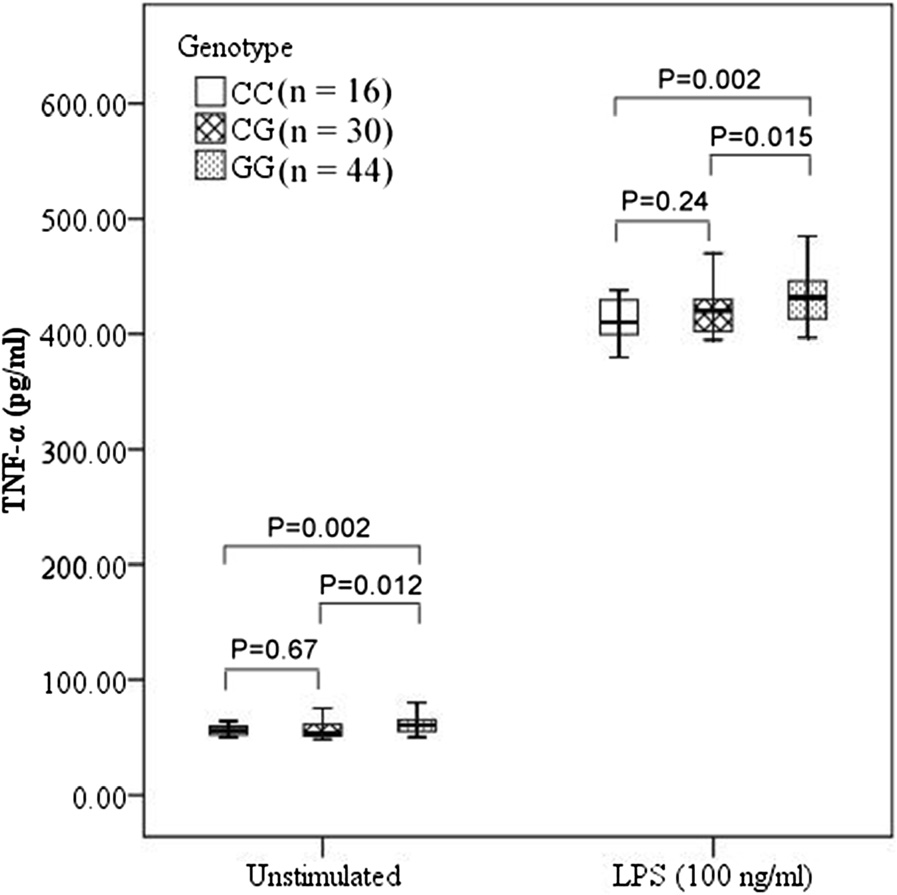
**Association of TNF-α levels and rs4755453 genotype.** Concentrations of TNF-α in culture supernatants were expressed as the median, interquartile range and extremes. The TNF-α levels were significantly different among GG, GC and CC genotypes both at baseline (*P* = 0.002) and under the LPS-stimulated condition (*P* = 0.003).

**Figure 3:**
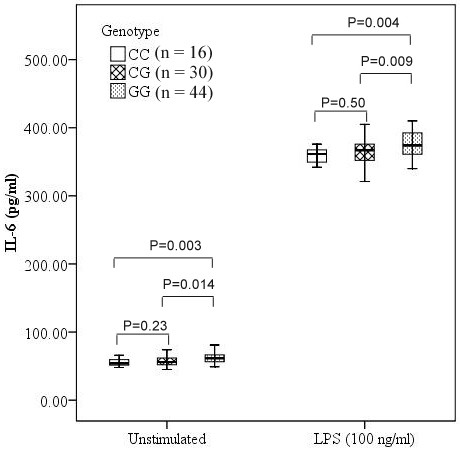
**Association of IL-6 levels and rs4755453 genotype.** Concentrations of IL-6 in culture supernatants were expressed as the median, interquartile range and extremes. The IL-6 levels were significantly different among GG, GC and CC genotypes both at baseline (*P* = 0.003) and under the LPS-stimulated condition (*P* = 0.003).

To determine whether rs4755453 genotypes influenced the inflammatory cytokine production, we investigated the TNF-α and IL-6 levels in cell culture supernatants of PBMCs. We observed a significant association between TNF-α and IL-6 levels and rs4755453 genotypes. Subjects with homozygotes for rs4755453G allele were associated with higher levels of TNF-α and IL-6 compared with heterozygotes and homozygotes for the rs4755453C allele both at baseline (*P* = 0.012 and *P* = 0.002 for TNF-α; *P* = 0.009 and *P* = 0.004 for IL-6, respectively) and after LPS stimulation (*P* = 0.015 and *P* = 0.002 for TNF-α; P = 0.014 and *P* = 0.003 for IL-6, respectively) (Figures 2 and 3).

## Discussion

To our knowledge, this was the first study to report the potential role for genetic variation of *TRAF6* with sepsis-induced ALI susceptibility in Chinese Han population. We identified an intronic SNP (rs4755453) strongly associated with the development of sepsis-induced ALI. Moreover, our functional results showed that rs4755453 was associated with the expression of *TRAF6* mRNA and the production of TNF-α and IL-6. However, we observed no associations between variants in these four genes and ALI mortality in this study. Taken together, our findings clearly demonstrated a genetic predisposition that greater *TRAF6* mRNA expression might increase susceptibility to sepsis-induced ALI in the presence of clinical risk factors.

TLRs recognized conserved PAMPs or DAMPs and then initiated innate immunity response, which contributed to the overwhelming proinflammatory cytokine generation. A growing body of literature implicated that TLRs and their downstream components played an important role in the pathogenesis of ALI [25,41]. TRAF6 played a critical role in the TLR-mediated signaling pathway [42]. Liu et al. found that TRAF6 knockdown resulted in reduced TNF-α and IL-6 mRNA expression and promoted cell survival upon LPS challenge in primary rat proximal renal tubular cells [24]. Two studies found that greater activation of TRAF6 led to significant increase of the cytokines production and induced the chronic lung injury [43,44]. Similarly, Imai and colleagues found that inactivation of TRAF6 in TRAF6^MC-KO^ mice alleviated the degree of ALI by inhibiting the expression of IL-6. And two experimental ALI models in their study also proved that innate immune signaling via TLR4-TRIF-TRAF6 was a key genetic pathway that determined the susceptibility to acute lung failure [26]. Although several variants in the TLR signaling pathway genes were implicated in susceptibility to ALI and sepsis, the effect of variation in *TRAF6* on human diseases susceptibility was not reported till now.

How might *TRAF6* variant (rs4755453) affect susceptibility to ALI? Our functional study showed that the risk allele (rs4755453G) was associated with increased *TRAF6* mRNA expression and TNF-α and IL-6 production at baseline and after innate immune stimulation with LPS. Therefore, it was possible that rs4755453 influenced the expression of *TRAF6* mRNA, and subsequently increased the production of inflammatory cytokines, which directly induced lung tissue injury. As an intron polymorphism, the exact mechanism of rs4755453 induced a phenotypic change was currently unclear. Rs4755453 is located in the first intron region of *TRAF6*. Whether this variation influences *TRAF6* mRNA stability and translation directly, induces exon skipping, enhances the use of cryptic splice sites or alters the ratio of alternatively spliced isoforms is needed to be investigated in future studies.

Rs1059703 (-1595C/T), tagged the *IRAK1* functional haplotype, was found to be associated with exaggerated NF-κB activation both *in vitro* and *in vivo*. In the Caucasian population, this functional haplotype was demonstrated to be associated with the severity of pulmonary injury, the risk of septic shock, higher mortality rate of sepsis and the need for prolonged mechanical ventilation [30,31]. However, in our data, rs1059703 was not associated with susceptibility to ALI, higher mortality rate and ventilator-free days even in the subgroup of patients aged < 65 years (data not shown). Two factors might contribute to such discrepancy. The allele and genotype distributions of rs1059703 were different between these two ethnicities. The risk C allele frequency of rs1059703 in Han Chinese descent (87.5%) from Hapmap data was significantly higher than that in Europeans descent (18.9%). Allele frequency might reflect the different natural selection or infection pressures [32]. In addition, clinical heterogeneity might also contribute to this discrepancy. The individuals served as cases in the two Caucasian studies were sepsis patients. However, the cases enrolled in our study were ALI patients caused by severe sepsis and septic shock.

Our study had several clear strengths. First, sepsis alone patients who did not develop ALI/ARDS were served as controls in our study. Such controls were preferable to healthy individuals since a proportion of healthy subjects might develop ALI/ARDS under the stimulus for lung injury. Second, to minimize racial admixture, we focused on central Han Chinese patients, which could be regarded as one single homogenous population. Of note, there were two limitations in the current study. First, although we have adequate power to detect rs4755453 association with sepsis induced-ALI using the current data, independent samples were still needed to validate the associations. Second, it was possible that rs4755453 serves as a marker for an as-yet unknown functional variant within the TRAF6 gene. Exhaustive re-sequencing should be performed to find or rule out the possibility of an as-yet-unidentified causal SNP in linkage disequilibrium with rs4755453.

## Conclusions

We reported for the first time that a tag SNP, in the intron region of *TRAF6*, was associated with sepsis-induced ALI susceptibility in Chinese Han population. These findings might have important implications in our understanding the pathophysiology of ALI and the role of genetic variation on the development of this lethal respiratory syndrome. However, as this was the first study to report the genetic variation in *TRAF6* and ALI risk, future studies were needed to validate the associations in other populations.

## Competing interests

The authors declare that they have no competing interests.

## Authors’ contributions

ZJS and CLY carried out the molecular genetic studies, participated in the sequence alignment and drafted the manuscript. JY carried out the immunoassays. JY, ZD, ZGT and SS participated in the sequence alignment. CYT, JY, ZS, DMZ, JJJ, YPZ and MS participated in the design of the study and performed the statistical analysis. CXB and ZJS conceived of the study, and participated in its design and coordination and helped to draft the manuscript. All authors read and approved the final manuscript.

## Supplementary Material

Additional file 1 **Table S1 **The primers of twelve tagSNPs in *IRAK1*, *IRAK4* and *TRAF6.* Table S2. Primers and PCR protocols for two tagSNPs in *MyD88.* Table S3. Association analysis of genetic variation in *TRAF6, MyD88, IRAK4* and *IRAK1* between survivors and non-survivors of ALI. Table S4. Association analysis of genetic variation in *TRAF6, MyD88, IRAK4* and *IRAK1* between ALI and ARDS patients. (DOC 300 kb) Click here for file
