# Supplementary material for: Genetic variation in the TNF receptor-associated factor 6 gene is associated with susceptibility to sepsis-induced acute lung injury
Source: J Transl Med. 2012 Aug 17;10:166. doi: 10.1186/1479-5876-10-166 (PMC3478205; doi:10.1186/1479-5876-10-166)
Supplement: Additional file 1 — Table S1 The primers of twelve tagSNPs in IRAK1, IRAK4 and TRAF6. Table S2. Primers and PCR protocols for two tagSNPs in MyD88. Table S3. Association analysis of genetic variation in TRAF6, MyD88, IRAK4 and IRAK1 between survivors and non-survivors of ALI. Table S4. Association analysis of genetic variation in TRAF6, MyD88, IRAK4 and IRAK1 between ALI and ARDS patients. (DOC 300 kb) [file 1479-5876-10-166-S1.doc]

**Supplementary table 1** The primers of twelve tagSNPs in *IRAK1*, *IRAK4* and *TRAF6*

| Gene | SNP | Oligo type-mode | Sequence |  | |  | |  | |  |  |  |
| --- | --- | --- | --- | --- | --- | --- | --- | --- | --- | --- | --- | --- |
| IRAK1 | rs1059703 | PCRU-1 | TGCTGGACACGTAGGAGTTCT | | | |  | |  | |  |  |
|  |  | PCRU-2 | TGCTGGACATGTAGGAGTTCT | | | |  | |  | |  |  |
|  |  | PCRL | CTAGAGAAGCTGCAGGCAGT | | | |  | |  | |  |  |
|  |  | SNPU | ACGCACGTCCACGGTGATTTAGGGGGGATGCAGCTGGCGGCCTCN | |  | | | | | | | |
| IRAK4 | rs1461567 | PCRU | TTTTATCAGAAACCCACTCCC |  | |  | |  | |  | | |
|  |  | PCRL | TAGTTAGGCCATGAGAGCTTTG |  | |  | |  | |  | | |
|  |  | SNPU | GCGGTAGGTTCCCGACATATGATAACTAACCCACTCCTGCCATGA | |  | | | | | | | |
| IRAK4 | rs4251569 | PCRU | TTGGCTTTGGTTAGAACTTAGG |  | |  | |  | |  | | |
|  |  | PCRL | CATTAGCACTTTGATCAGAGGAA |  | |  | |  | |  | | |
|  |  | SNPU | AGCGATCTGCGAGACCGTATCTGTGCAACCTCAAAGTTCATCTTC | |  | | | | | | | |
| IRAK4 | rs4251466 | PCRU | ATGTGTGTGCATCCTATAACCC |  | |  | |  | |  | | |
|  |  | PCRL | TACAACATTAAAGAAAAGGGACAAC |  | |  | |  | |  | | |
|  |  | SNPU | GGATGGCGTTCCGTCCTATTTTTTCCTCCTATCTTTCCTGTCTCT | |  | | | | | | | |
| IRAK4 | rs4251429 | PCRU | TTTACCTTTCTGACCTCATCTGC |  | |  | |  | |  | | |
|  |  | PCRL_1 | AAAAGTATGTGGACAAAACGAA |  | |  | |  | |  | | |
|  |  | PCRL_2 | AAAAGTTTGTGGACAAAACGAA |  | |  | |  | |  | | |
|  |  | SNPU | GTGATTCTGTACGTGTCGCCTCCTTGCTGTTGCCCAGTGGGCCCA | |  | | | | | | | |
| IRAK4 | rs4251545 | PCRU | AAGATGAAGAAAAGACAATTGAAGAT |  | |  | |  | |  | | |
|  |  | PCRL | TATTTTTCTTTTCATGCAGACATT |  | |  | |  | |  | | |
|  |  | SNPU | GGCTATGATTCGCAATGCTTTGATGCTGATTCCACTTCAGTTGAA | |  | | | | | | | |
| IRAK4 | rs4251513 | PCRU | ATTAAGCTGTGCTTATGTGCCT |  | |  | |  | |  | | |
|  |  | PCRL | TCAGTAACCCTACTGAACACATCA |  | |  | |  | |  | | |
|  |  | SNPU | ACGCACGTCCACGGTGATTTGATCCAGATTAAAAGGGAGAGGATA | |  | | | | | | | |
| IRAK4 | rs4251431 | PCRU | ATACTATAGAGTTTATTTATTTGCTTATTGCC | | |  | |  | |  | | |
|  |  | PCRL | TAGATACTGCTCCAGGCCC | | | |  | |  | |  |  |
|  |  | SNPU | AGGGTCTCTACGCTGACGATTGAATTCAGGTTTTGGTTTTGTTTT | |  | | | | | | | |
| IRAK4 | rs3794262 | PCRU | CCATCAGGGTAGAAGTTGAA |  | |  | |  | |  | | |
|  |  | PCRL | TAGTTTTGTGCCAACAGGAAG |  | |  | |  | |  | | |
|  |  | SNPU | GGATGGCGTTCCGTCCTATTTTACTTTCTTACAGCCTAAGCCAGA | |  | | | | | | | |
| TRAF-6 | rs540386 | PCRU | AAACAATGGCAAGACAGCA | | | |  | |  | |  |  |
|  |  | PCRL | TCAGATTTGGAAGGTATAAAGGG |  | |  | |  | |  | | |
|  |  | SNPU | GGCTATGATTCGCAATGCTTGCAGAACTAGTCACTACAGCTGGGT | |  | | | | | | | |
| TRAF-6 | rs4755453 | PCRU | TGCCTATACACTCAACCTCAAA |  | |  | |  | |  | | |
|  |  | PCRL | TTGGCCTTGTGAACTTGG | | | |  | |  | |  |  |
|  |  | SNPU | AGCGATCTGCGAGACCGTATAAGCCCCTCTCCCTTCCCTAGTAAT | |  | | | | | | | |
| TRAF-6 | rs5030493 | PCRU | AAATTGCCTCCTGCAGTTG | | | |  | |  | |  |  |
|  |  | PCRL_1 | ACAGGGACAGCCGGAGTC | | | |  | |  | |  |  |
|  |  | PCRL_2 | ACAGTGACAGCCGGAGTC | | | |  | |  | |  |  |
|  |  | SNPU | AGATAGAGTCGATGCCAGCTGCTCCTTGGAGCAAACACCTGCTTA |  | |  | |  | |  |  |  |

SNP, single nucleotide polymorphism

Note: Primer design for PCR and single base extension (SBE) was performed with Beckman Coulter Autoprimer software. PCR reactions in the first step were composed of PCR primers (PCRU and PCRL) at a final concentration of 50 nM, 0.2 U of enzyme Hot Master Taq (Eppendorf, Hamburg, Germany), and 10 ng of genomic DNA per reaction. PCR products were used as template for the single base extension (SBE). SBE was performed using SBE primers (SNPU) and reagents specific to the SNPstream platform. SBE reaction products were hybridized to Beckman array plates and scanned by the SNPstream.

**Supplementary table 2** Primers and PCR protocols for two tagSNPs in *MyD88*

| Gene | Target Primer | Sequence (5'→3') | PCR protocol |
| --- | --- | --- | --- |
| MyD88 | rs6853 |  | 95°C for 5 min; 35 cycles at 94°C for 30 s, 57°C for 40 s, |
|  |  |  | and 72°C for 45 s; followed by 72°C for 10 min |
|  | Forward | GCACATGGGCACATACAGAC |  |
|  | Reverse | CCAGTGGACCAGCTTCTCTT |  |
|  | rs7744 |  | 95°C for 5 min; 35 cycles at 94°C for 30 s, 57°C for 40 s, |
|  | Forward | GCACATGGGCACATACAGAC | and 72°C for 45 s; followed by 72°C for 10 min |
|  | Reverse | ATATGCAGCCTCCGGATTGT |  |

**Supplementary table 3** Association analysis of genetic variation in *TRAF6, MyD88, IRAK4* and *IRAK1* between survivors and non-survivors of ALI

|  |  |  |  | Allelic Comparison | | | | Genotypic Comparison | |
| --- | --- | --- | --- | --- | --- | --- | --- | --- | --- |
| Gene | SNP | Survior | Nonsurvior | *P* | *P*_adj_ | OR (95% CI) | OR_adj_ (95% CI) | *P* | *P*_adj_ |
| TRAF6 | rs540386 |  |  | 0.65 | 0.69 | 0.80 (0.30 -2.11) | 0.83 (0.29 -1.94) | 0.65 | 0.67 |
|  | CC | 118 (92.9%) | 132 (94.3%) |  |  |  |  |  |  |
|  | TC | 9 (7.1%) | 8 (5.7%) |  |  |  |  |  |  |
|  | C | 245 (96.5%) | 272 (97.1%) |  |  |  |  |  |  |
|  | T | 9 (3.5%) | 8 (2.9%) |  |  |  |  |  |  |
|  | rs4755453 |  |  | 0.40 | 0.43 | 0.79 (0.45 -1.38) | 0.63 (0.42 - 1.38) | 0.72 | 0.79 |
|  | GG | 99 (78%) | 116 (82.3%) |  |  |  |  |  |  |
|  | CG | 27 (21.3%) | 24 (17%) |  |  |  |  |  |  |
|  | CC | 1 (0.8%) | 1 (0.7%) |  |  |  |  |  |  |
|  | G | 225 (88.6%) | 256 (90.8%) |  |  |  |  |  |  |
|  | C | 29 (11.4%) | 26 (9.2%) |  |  |  |  |  |  |
|  | rs5030493 |  |  | 0.47 | 0.36 | 1.25 (0.68 -2.27) | 1.28 (0.54 - 2.45) | 0.45 | 0.38 |
|  | AA | 104 (83.9%) | 114 (80.3%) |  |  |  |  |  |  |
|  | TA | 20 (16.1%) | 28 (19.7%) |  |  |  |  |  |  |
|  | A | 228 (91.9%) | 256 (90.1%) |  |  |  |  |  |  |
|  | T | 20 (8.1%) | 28 (9.9%) |  |  |  |  |  |  |
| MyD88 | rs6843 |  |  | 0.18 | 0.34 | 2.02 (0.69-5.90) | 1.94 (0.68-4.82) | 0.18 | 0.22 |
|  | AA | 123 (96.1%) | 131 (92.3%) |  |  |  |  |  |  |
|  | AG | 5 (3.9%) | 11 (7.7%) |  |  |  |  |  |  |
|  | A | 251 (98%) | 273 (96.1%) |  |  |  |  |  |  |
|  | G | 5 (2%) | 11 (3.9%) |  |  |  |  |  |  |
|  | rs7744 |  |  | 0.05 | 0.19 | 1.42 (1.01-2.01) | 1.28 (0.98-1.96) | 0.11 | 0.29 |
|  | AA | 48 (37.8%) | 39 (27.7%) |  |  |  |  |  |  |
|  | AG | 66 (52%) | 78 (55.3%) |  |  |  |  |  |  |
|  | GG | 13 (10.2%) | 24 (17%) |  |  |  |  |  |  |
|  | A | 162 (63.8%) | 156 (55.3%) |  |  |  |  |  |  |
|  | G | 92 (36.2%) | 126 (44.7%) |  |  |  |  |  |  |
| IRAK4 | rs3794262 |  |  | 0.70 | 0.42 | 0.91 (0.58-1.44) | 0.78 (0.82-1.25) | 0.88 | 0.49 |
|  | AA | 85 (66.9%) | 97 (69.3%) |  |  |  |  |  |  |
|  | TA | 40 (31.5%) | 41 (29.3%) |  |  |  |  |  |  |
|  | TT | 2 (1.6%) | 2 (14.3%) |  |  |  |  |  |  |
|  | A | 210 (82.7%) | 235 (83.9%) |  |  |  |  |  |  |
|  | T | 44 (17.3%) | 45 (16.1%) |  |  |  |  |  |  |
|  | rs4251429 |  |  | 0.06 | 0.28 | 1.96 (0.97-3.99) | 1.56 (0.78-2.56) | 0.05 | 0.22 |
|  | GG | 116 (90.6%) | 117 (82.4%) |  |  |  |  |  |  |
|  | GC | 12 (9.4%) | 25 (17.6%) |  |  |  |  |  |  |
|  | G | 244 (95.3%) | 259 (91.2%) |  |  |  |  |  |  |
|  | C | 12 (4.7%) | 25 (8.8%) |  |  |  |  |  |  |
|  | rs4251545 |  |  | 0.64 | 0.72 | 0.88 (0.50-1.52) | 0.91(0.73-1.34) | 0.10 | 0.21 |
|  | GG | 98 (77.8%) | 117 (82.4%) |  |  |  |  |  |  |
|  | GA | 28 (22.2%) | 22 (15.5%) |  |  |  |  |  |  |
|  | AA | 0 | 3 (2.1%) |  |  |  |  |  |  |
|  | G | 224 (88.9%) | 256 (90.1%) |  |  |  |  |  |  |
|  | A | 28 (11.1%) | 28 (9.9%) |  |  |  |  |  |  |
|  | rs4251569 |  |  |  |  |  |  |  |  |
|  | CC | 95 (75.4%) | 105 (73.9%) | 0.92 | 0.82 | 1.03 (0.62-1.69) | 1.06 (0.78-1.78) | 0.75 | 0.62 |
|  | CT | 29 (23%) | 36 (25.4%) |  |  |  |  |  |  |
|  | TT | 2 (1.6%) | 1 (0.7%) |  |  |  |  |  |  |
|  | C | 219 (86.9%) | 246 (86.6%) |  |  |  |  |  |  |
|  | T | 33 (13.1%) | 38 (13.4%) |  |  |  |  |  |  |
|  | rs4251513 |  |  |  |  |  |  |  |  |
|  | CC | 55 (43%) | 56 (40%) | 0.98 | 0.78 | 1.00 (0.71-1.43) | 1.08 (0.83-1.52) | 0.63 | 0.44 |
|  | CG | 53 (41.4%) | 66 (47.1%) |  |  |  |  |  |  |
|  | GG | 20 (15.6%) | 18 (12.9%) |  |  |  |  |  |  |
|  | C | 163 (63.7%) | 178 (63.6%) |  |  |  |  |  |  |
|  | G | 93 (36.3%) | 102 (36.4%) |  |  |  |  |  |  |
|  | rs4251466 |  |  |  |  |  |  |  |  |
|  | CC | 101 (80.2%) | 115 (81%) | 0.75 | 0.46 | 0.91 (0.52-1.61) | 1.02 (0.78-1.87) | 0.81 | 0.66 |
|  | CT | 24 (19%) | 27 (19%) |  |  |  |  |  |  |
|  | TT | 1 (0.8%) | 0 |  |  |  |  |  |  |
|  | C | 226 (89.7%) | 257 (90.5%) |  |  |  |  |  |  |
|  | T | 26 (10.3%) | 27 (9.5%) |  |  |  |  |  |  |
|  | rs4251431 |  |  |  |  |  |  |  |  |
|  | GG | 100 (79.4%) | 121 (85.2%) | 0.30 | 0.18 | 0.73 (0.40-1.32) | 0.87 (0.56-1.45) | 0.19 | 0.11 |
|  | GT | 26 (20.6%) | 20 (14.1%) |  |  |  |  |  |  |
|  | TT | 0 | 1 (0.7%) |  |  |  |  |  |  |
|  | G | 226 (89.7%) | 262 (92.3%) |  |  |  |  |  |  |
|  | T | 26 (10.3%) | 22 (7.7%) |  |  |  |  |  |  |
|  | rs1461567 |  |  |  |  |  |  |  |  |
|  | CC | 42 (33.6%) | 46 (32.4%) | 0.59 | 0.81 | 1.10 (0.78-1.55) | 1.02 (0.56-1.38) | 0.81 | 0.68 |
|  | TC | 53 (42.4%) | 57 (40.1%) |  |  |  |  |  |  |
|  | TT | 30 (24%) | 39 (27.5%) |  |  |  |  |  |  |
|  | C | 137 (54.8%) | 149 (52.5%) |  |  |  |  |  |  |
|  | T | 113 (45.2%) | 135 (47.5%) |  |  |  |  |  |  |
| IRAK1 | rs1059703 |  |  | 0.41 | 0.57 | 0.61 (0.53-1.56) ^1^ | 0.69 (0.64-1.68) | - | - |
|  | CC/C- | 103 (81.1%) | 117 (84.2%) |  |  |  |  |  |  |
|  | CT | 19 (15%) | 19 (13.7%) |  |  |  |  |  |  |
|  | TT/T- | 5 (3.9%) | 3 (2.2%) |  |  |  |  |  |  |

SNP, single nucleotide polymorphism; ALI, acute lung injury; OR, odds ratio; CI, confidence interval.

*P* was determined using the chi-square test. *P_adj_* and OR_adj_ came from multivariate logistic regression.

A *P*-value of < 0.0036 (0.05/14) was considered statistically significant after Bonferroni correction.

^1^ The p value was calculated using logistic regression method including sex as a covariate, in which males were coded as homozygote females.

**Supplementary table 4** Association analysis of genetic variation in *TRAF6, MyD88, IRAK4* and *IRAK1* between ALI and ARDS patients

|  |  |  |  | Allelic Comparison | | | | Genotypic Comparison | |
| --- | --- | --- | --- | --- | --- | --- | --- | --- | --- |
| Gene | SNP | ALI | ARDS | *P* | *P*_adj_ | OR (95% CI) | OR_adj_ (95% CI) | *P* | *P*_adj_ |
| TRAF6 | rs540386 |  |  | 0.27 | 0.41 | 0.57 (0.21-1.56) | 0.62 (0.32-1.47) | 0.26 | 0.34 |
|  | CC | 58 (90.6%) | 192 (94.6%) |  |  |  |  |  |  |
|  | TC | 6 (9.4%) | 11 (5.4%) |  |  |  |  |  |  |
|  | C | 122 (95.3%) | 395 (97.3%) |  |  |  |  |  |  |
|  | T | 6 (4.7%) | 11 (2.7%) |  |  |  |  |  |  |
|  | rs4755453 |  |  | 0.30 | 0.19 | 0.72 (0.39-1.34) | 0.68 (0.38-1.31) | 0.15 | 0.24 |
|  | GG | 48 (76.2%) | 167 (81.5%) |  |  |  |  |  |  |
|  | CG | 14 (22.2%) | 37 (18%) |  |  |  |  |  |  |
|  | CC | 1 (1.6%) | 1 (0.5%) |  |  |  |  |  |  |
|  | G | 110 (87.3%) | 371 (90.5%) |  |  |  |  |  |  |
|  | C | 16 (12.7%) | 39 (9.5%) |  |  |  |  |  |  |
|  | rs5030493 |  |  | 0.90 | 0.76 | 1.05 (0.52-2.12) | 1.12 (0.42-2.26) | 0.89 | 0.73 |
|  | AA | 52 (82.5%) | 166 (81.8%) |  |  |  |  |  |  |
|  | TA | 11 (17.5%) | 37 (18.2%) |  |  |  |  |  |  |
|  | A | 115 (91.3%) | 369 (90.9%) |  |  |  |  |  |  |
|  | T | 11 (8.7%) | 37 (9.1%) |  |  |  |  |  |  |
| MyD88 | rs6843 |  |  | 0.16 | 0.32 | 3.13 (0.70-13.9) | 2.97 (0.63-8.95) | 0.37 | 0.42 |
|  | AA | 62 (96.9%) | 192 (93.2%) |  |  |  |  |  |  |
|  | AG | 2 (3.1%) | 14 (6.8%) |  |  |  |  |  |  |
|  | A | 162 (98.8%) | 362 (96.3%) |  |  |  |  |  |  |
|  | G | 2 (1.2%) | 14 (3.7%) |  |  |  |  |  |  |
|  | rs7744 |  |  | 0.21 | 0.32 | 1.30 (0.86-1.96) | 1.24 (0.93-1.65) | 0.28 | 0.39 |
|  | AA | 26 (40.6%) | 61 (29.9%) |  |  |  |  |  |  |
|  | AG | 30 (46.9%) | 114 (55.9%) |  |  |  |  |  |  |
|  | GG | 8 (12.5%) | 29 (14.2%) |  |  |  |  |  |  |
|  | A | 82 (64.1%) | 236 (57.8%) |  |  |  |  |  |  |
|  | G | 46 (35.9%) | 172 (42.2%) |  |  |  |  |  |  |
| IRAK4 | rs3794262 |  |  | 0.93 | 0.76 | 1.03 (0.60-1.75) | 1.12 (0.54-1.89) | 0.68 | 0.52 |
|  | AA | 43 (67.2%) | 139 (68.5%) |  |  |  |  |  |  |
|  | TA | 21 (32.8%) | 60 (29.6%) |  |  |  |  |  |  |
|  | TT | 0 | 4 (2%) |  |  |  |  |  |  |
|  | A | 107 (83.6%) | 338 (83.3%) |  |  |  |  |  |  |
|  | T | 21 (16.4%) | 68 (16.7%) |  |  |  |  |  |  |
|  | rs4251429 |  |  | 0.93 | 0.98 | 0.96 (0.44-2.10) | 0.98 (0.67-1.94) | 0.93 | 0.96 |
|  | GG | 55 (85.9%) | 178 (86.4%) |  |  |  |  |  |  |
|  | GC | 9 (14.1%) | 28 (13.6%) |  |  |  |  |  |  |
|  | G | 119 (93%) | 384 (93.2%) |  |  |  |  |  |  |
|  | C | 9 (7%) | 28 (6.8%) |  |  |  |  |  |  |
|  | rs4251545 |  |  | 0.78 | 0.54 | 0.91 (0.48-1.73) | 0.65 (0.55-1.56) | 0.87 | 0.76 |
|  | GG | 50 (79.4%) | 165 (80.5%) |  |  |  |  |  |  |
|  | GA | 12 (19%) | 38 (18.5%) |  |  |  |  |  |  |
|  | AA | 1 (1.6%) | 2 (1%) |  |  |  |  |  |  |
|  | G | 112 (88.9%) | 368 (89.8%) |  |  |  |  |  |  |
|  | A | 14 (11.1%) | 42 (10.2%) |  |  |  |  |  |  |
|  | rs4251569 |  |  | 0.84 | 0.92 | 1.07 ( 0.59-1.94) | 1.02 (0.87-1.56) | 0.11 | 0.32 |
|  | CC | 49 (77.8%) | 151 (73.7%) |  |  |  |  |  |  |
|  | CT | 12 (19%) | 53 (25.8%) |  |  |  |  |  |  |
|  | TT | 2 (3.2%) | 1 (0.5%) |  |  |  |  |  |  |
|  | C | 110 (87.3%) | 355 (86.6%) |  |  |  |  |  |  |
|  | T | 16 (12.7%) | 55 (13.4%) |  |  |  |  |  |  |
|  | rs4251513 |  |  | 0.25 | 0.32 | 0.79 (0.53-1.19) | 0.84 (0.76-1.47) | 0.48 | 0.53 |
|  | CC | 24 (37.5%) | 87 (42.7%) |  |  |  |  |  |  |
|  | CG | 28 (43.8%) | 91 (44.6%) |  |  |  |  |  |  |
|  | GG | 12 (18.7%) | 26 (12.7%) |  |  |  |  |  |  |
|  | C | 76 (59.4%) | 265 (65%) |  |  |  |  |  |  |
|  | G | 52 (40.6%) | 143 (35%) |  |  |  |  |  |  |
|  | rs4251466 |  |  | 0.09 | 0.12 | 0.59 (0.32-1.09) | 0.61 (0.38-1.12) | 0.07 | 0.17 |
|  | CC | 45 (73.8%) | 171 (82.6%) |  |  |  |  |  |  |
|  | CT | 15 (24.6%) | 36 (17.4%) |  |  |  |  |  |  |
|  | TT | 1 (1.6%) | 0 |  |  |  |  |  |  |
|  | C | 105 (86.1%) | 378 (91.3%) |  |  |  |  |  |  |
|  | T | 17 (13.9%) | 36 (8.7%) |  |  |  |  |  |  |
|  | rs4251431 |  |  | 0.60 | 0.65 | 1.21 (0.59-2.51) | 1.18 (0.62-1.63) | 0.89 | 0.76 |
|  | GG | 54 (84.4%) | 167 (81.9%) |  |  |  |  |  |  |
|  | GT | 10 (15.6%) | 36 (17.6%) |  |  |  |  |  |  |
|  | TT | 0 | 1 (0.5%) |  |  |  |  |  |  |
|  | G | 118 (92.2%) | 370 (90.7%) |  |  |  |  |  |  |
|  | T | 10 (7.8%) | 38 (9.3%) |  |  |  |  |  |  |
|  | rs1461567 |  |  | 0.92 | 0.59 | 1.02 (0.68-1.53) | 1.09 (0.68-1.56) | 0.51 | 0.28 |
|  | CC | 23 (36.5%) | 65 (31.9%) |  |  |  |  |  |  |
|  | TC | 22 (34.9%) | 88 (43.1%) |  |  |  |  |  |  |
|  | TT | 18 (28.6%) | 51 (25%) |  |  |  |  |  |  |
|  | C | 68 (54%) | 218 (53.4%) |  |  |  |  |  |  |
|  | T | 58 (46%) | 190 (46.6%) |  |  |  |  |  |  |
| IRAK1 | rs1059703 |  |  | 0.47 | 0.59 | 0.65 (0.61 -1.74) ^1^ | 0.72 (0.86 -1.61) | - | - |
|  | CC/C- | 51 (81%) | 169 (83.3%) |  |  |  |  |  |  |
|  | CT | 10 (15.9%) | 28 (13.8%) |  |  |  |  |  |  |
|  | TT/T- | 2 (3.1%) | 6 (2.9%) |  |  |  |  |  |  |

SNP, single nucleotide polymorphism; ALI, acute lung injury; ARDS, acute respiratory distress syndrome; OR, odds ratio; CI, confidence interval. Data were no. (%) of subjects. *P* was determined using the chi-square test. *P_adj_* and OR_adj_ came from multivariate logistic regression.

A *P*-value of < 0.0036 (0.05/14) was considered statistically significant after Bonferroni correction.

^1^ The p value was calculated using logistic regression method including sex as a covariate, in which males were coded as homozygote females.
